# The evolving role of morphology in endometrial cancer diagnostics: From histopathology and molecular testing towards integrative data analysis by deep learning

**DOI:** 10.3389/fonc.2022.928977

**Published:** 2022-08-18

**Authors:** Sarah Fremond, Viktor Hendrik Koelzer, Nanda Horeweg, Tjalling Bosse

**Affiliations:** ^1^ Department of Pathology, Leiden University Medical Center (LUMC), Leiden, Netherlands; ^2^ Department of Pathology and Molecular Pathology, University Hospital and University of Zürich, Zürich, Switzerland; ^3^ Department of Radiotherapy, Leiden University Medical Center, Leiden, Netherlands

**Keywords:** endometrial carcinoma, tumour morphology, computer vision, deep learning, molecular classification, phenotype, whole slide image, histopathology image

## Abstract

Endometrial cancer (EC) diagnostics is evolving into a system in which molecular aspects are increasingly important. The traditional histological subtype-driven classification has shifted to a molecular-based classification that stratifies EC into *DNA polymerase epsilon* mutated (*POLE*mut), mismatch repair deficient (MMRd), and p53 abnormal (p53abn), and the remaining EC as no specific molecular profile (NSMP). The molecular EC classification has been implemented in the World Health Organization 2020 classification and the 2021 European treatment guidelines, as it serves as a better basis for patient management. As a result, the integration of the molecular class with histopathological variables has become a critical focus of recent EC research. Pathologists have observed and described several morphological characteristics in association with specific genomic alterations, but these appear insufficient to accurately classify patients according to molecular subgroups. This requires pathologists to rely on molecular ancillary tests in routine workup. In this new era, it has become increasingly challenging to assign clinically relevant weights to histological and molecular features on an individual patient basis. Deep learning (DL) technology opens new options for the integrative analysis of multi-modal image and molecular datasets with clinical outcomes. Proof-of-concept studies in other cancers showed promising accuracy in predicting molecular alterations from H&E-stained tumor slide images. This suggests that some morphological characteristics that are associated with molecular alterations could be identified in EC, too, expanding the current understanding of the molecular-driven EC classification. Here in this review, we report the morphological characteristics of the molecular EC classification currently identified in the literature. Given the new challenges in EC diagnostics, this review discusses, therefore, the potential supportive role that DL could have, by providing an outlook on all relevant studies using DL on histopathology images in various cancer types with a focus on EC. Finally, we touch upon how DL might shape the management of future EC patients.

## Introduction

**Figure 1:**
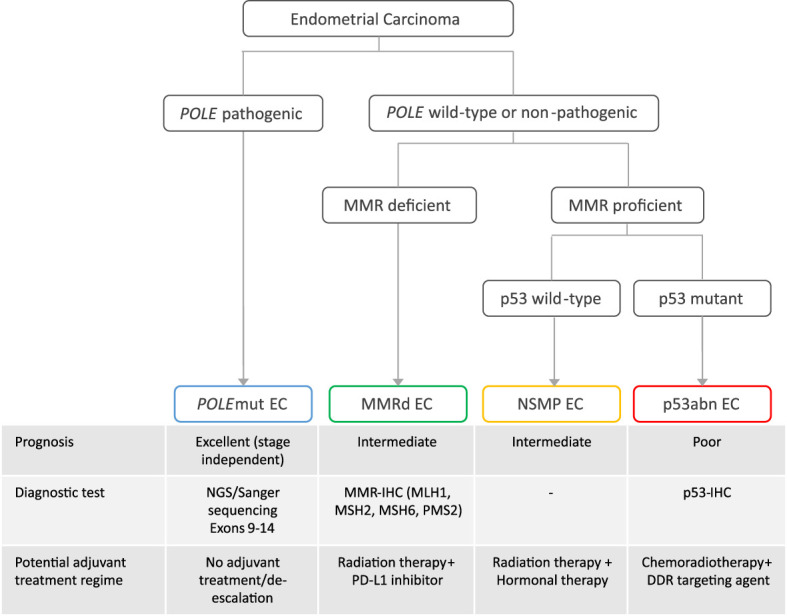
The diagnostic algorithm of the molecular classification of endometrial cancer, associated prognosis, diagnostic test, and potential adjuvant treatment regime. EC, endometrial cancer; NGS, panel next-generation sequencing; *POLE*mut, polymerase epsilon mutated; MMRd, mismatch repair deficient; NSMP, no specific molecular profile; p53abn, p53 abnormal; DDR, DNA damage response; PD-L1, programmed death ligand.

The incorporation of the molecular endometrial cancer (EC) classification in the fifth edition of the World Health Organization (WHO) classification of female genital tumors and the 2021 European treatment guidelines (1, 2) has marked a new era in EC diagnostics. This moved the field farther away from the classic dualistic model proposed by Bockman in 1983 (3), who divided endometrial carcinomas into type I and type II cancers. Type I EC is endometrioid and estrogen driven and can be graded using the International Federation of Gynaecology and Obstetrics (FIGO) grading system (4). Type II EC includes a variety of non-endometrioid histological subtypes, such as uterine serous carcinoma, clear cell carcinoma, mixed carcinomas, un-/dedifferentiated carcinomas, and uterine carcinosarcomas. The new molecular EC classification that is now recommended by the WHO (1, 2) completely changes the diagnostic perspective by placing histological subtype secondary to molecular class. It utilizes surrogate markers paralleling the four molecular classes originally described by The Cancer Genome Atlas (TCGA) (5). First, targeted sequencing (Sanger or panel next-generation sequencing, NGS) of exons 9–14 of *DNA polymerase epsilon* (*POLE*) can identify *POLE-*mutated (*POLE*mut) EC. Second, mismatch repair-deficient (MMRd) EC is defined by loss of expression of one of the mismatch repair proteins (MLH1, PMS2, MSH6, and MSH2) by immunohistochemistry (IHC). Third, p53 IHC is performed to identify EC with abnormal p53 expression patterns (p53abn) (6, 7). Finally, EC without a pathogenic *POLE* variant, with retained MMR protein expression, and wild-type p53 IHC, is classified as “no specific molecular profile” (NSMP) EC. Studies on EC with more than one molecular alteration, commonly referred to as “multiple-classifiers,” have served to identify the order by which these tests should be performed (8). It has resulted in the EC diagnostic algorithm endorsed by the WHO 2020 classification that first assesses *POLE* status regardless of other markers, then MMR, and finally p53 (9) (**Figure 1**).

The molecular classification resolves one of the main issues of the histology-driven EC classification, which is the high level of interobserver variability (10). Particularly high-grade and non-endometrioid histological subtypes are only moderately reproducible (11), which provides a poor basis for patient management (12). The recent paradigm shift in EC diagnostics follows preceding developments in surgical pathology, in which a series of technological breakthroughs such as immunohistochemistry and molecular testing have continually improved diagnostic accuracy (13).

The molecular EC classification has also shown a prognostic value across cohorts of different risk populations and is predictive of response to treatment, specifically in p53abn EC, which has a poor outcome and may benefit from addition of adjuvant chemotherapy (14), and in *POLE*mut EC, which has an excellent outcome regardless of adjuvant treatment, whereas MMRd and NSMP EC have intermediate prognoses (5, 14–20). This has been the rationale for adapting the clinical risk stratification system of EC patients (21) wherein it is encouraged to apply the molecular classification in the management of EC, especially high-risk EC (1, 2); ongoing and new trials such as PORTEC-4a (22) and RAINBO (23) exploit the molecular classification as a basis for targeted adjuvant therapy (**Figure 1**) (24). Consequently, the gynecological oncology community has started to utilize the molecular classification; however, the current risk stratification system does not clearly indicate which of the histological or molecular variables are most clinically relevant, or leverage the combination of both.

New technological breakthroughs in pathology are now driving progress in cancer diagnostics. Since the emergence of convolutional neural networks in 2012 (25), deep learning (DL) models have continuously demonstrated their high accuracy for the classification of medical (26) and non-medical image datasets (27). This was followed by the start of a digital revolution in pathology, wherein state-of-the-art DL models from the computer vision community were used on digital histopathology slides. Hematoxylin and eosin (H&E) staining procedure is the most common in cancer diagnostics, and large, well-characterized retrospective cohorts and clinical trial sets are available, enabling the collection of large-scale histopathology imaging datasets to train state-of-the-art DL models. A number of proof-of-concept papers showed the potential of DL models to aid the diagnosis and molecular classification of cancers (28–55) or predicting patient prognosis (56–60), by recognizing phenotypes on H&E-stained tumor slide images. Image-based DL models have been frequently trained onto colorectal cancer (29–37) and breast cancer (35, 38–44). However, in EC, only two studies (53, 54) so far have been published using DL for predicting one to various EC molecular alterations or gene mutations from publicly available datasets. They have obtained promising performance; however, the size of the dataset and application to a few non-endometrioid histological EC subtypes limit the generalizability of the findings. Furthermore, in these studies, DL models have not been trained to predict the newly established four-class molecular classification in EC diagnostics. Opportunities for future image-based DL models to impact EC diagnostics and thus guide clinical management decisions include the following: improve EC diagnostic classification by serving as a pre-screening tool to prioritize molecular testing, refine EC risk stratification by identifying morphological features with prognostic relevance, and predict patient outcome or even response to treatment.

**Figure 2:**
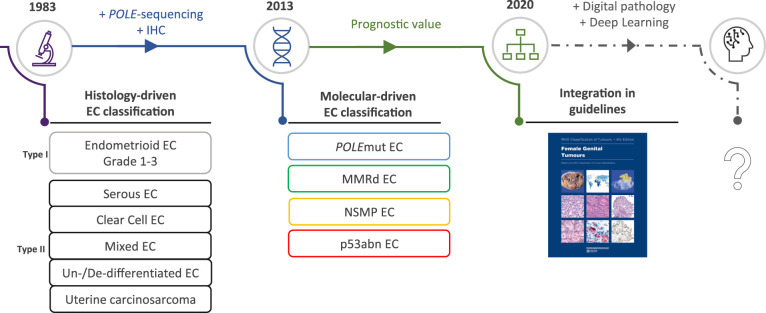
The evolving role of morphology in endometrial cancer diagnostics. EC, endometrial cancer; *POLE*mut, polymerase epsilon mutated; MMRd, mismatch repair deficient; NSMP, no specific molecular profile; p53abn, p53 abnormal.

In the light of the redefinition of EC on the basis of molecular features, we here provide a concise summary of the histopathological features associated with the four molecular classes (**Table 1**). These morpho-molecular correlates may serve to explore the feasibility of histology-directed molecular testing, particularly in low-income countries, and deepen our understanding of the underlying biological processes. We also present possible avenues by which image-based DL may be able to support these objectives, by discussing the landmark studies that have used DL onto histopathology slide images in EC and other cancers, which illustrates how these novel DL applications may impact the field of EC diagnostics in the future (**Figure 2**).

**Table 1:** Summary of the histopathological and immunohistochemical features correlated with the molecular endometrial cancer (EC) classification, dividing EC into *POLE*-mutated (*POLE*mut) EC, mismatch repair deficient (MMRd) EC, p53 abnormal (p53abn) EC, and non-specific molecular profile (NSMP) EC.

	*POLE*mut EC	MMRd EC	p53abn EC	NSMP EC
**Prototypical histopathological features**				
Glands with smooth luminal borders	++	+	−	+++
Glands with hobnailing (ragged luminal surface)	−	+	+++	−
Solid growth (at least 50%)	+++	++	+	+
Squamous differentiation (including morulae)	+	+	−	+++
Nuclear atypia	++	+	+++	+
Tumor giant cells	+++	−	+	−
Peri-tumoral and intra-epithelial infiltrate of lymphocytes	+++	++	−	−
Tertiary lymphoid structures (TLS)	+++	++	+	+
Microcystic elongated and fragmented (MELF)	−	+	−	++
Lymphovascular space invasion (LVSI)	+	++	+	+
				
**Immunohistochemical features**				
Abnormal MMR staining	+	+++	−	−
Abnormal p53 staining	+	+	+++	−

## Morpho-molecular correlates of the current EC classification

### *POLE-*mutated endometrial cancers

**Figure 3:**
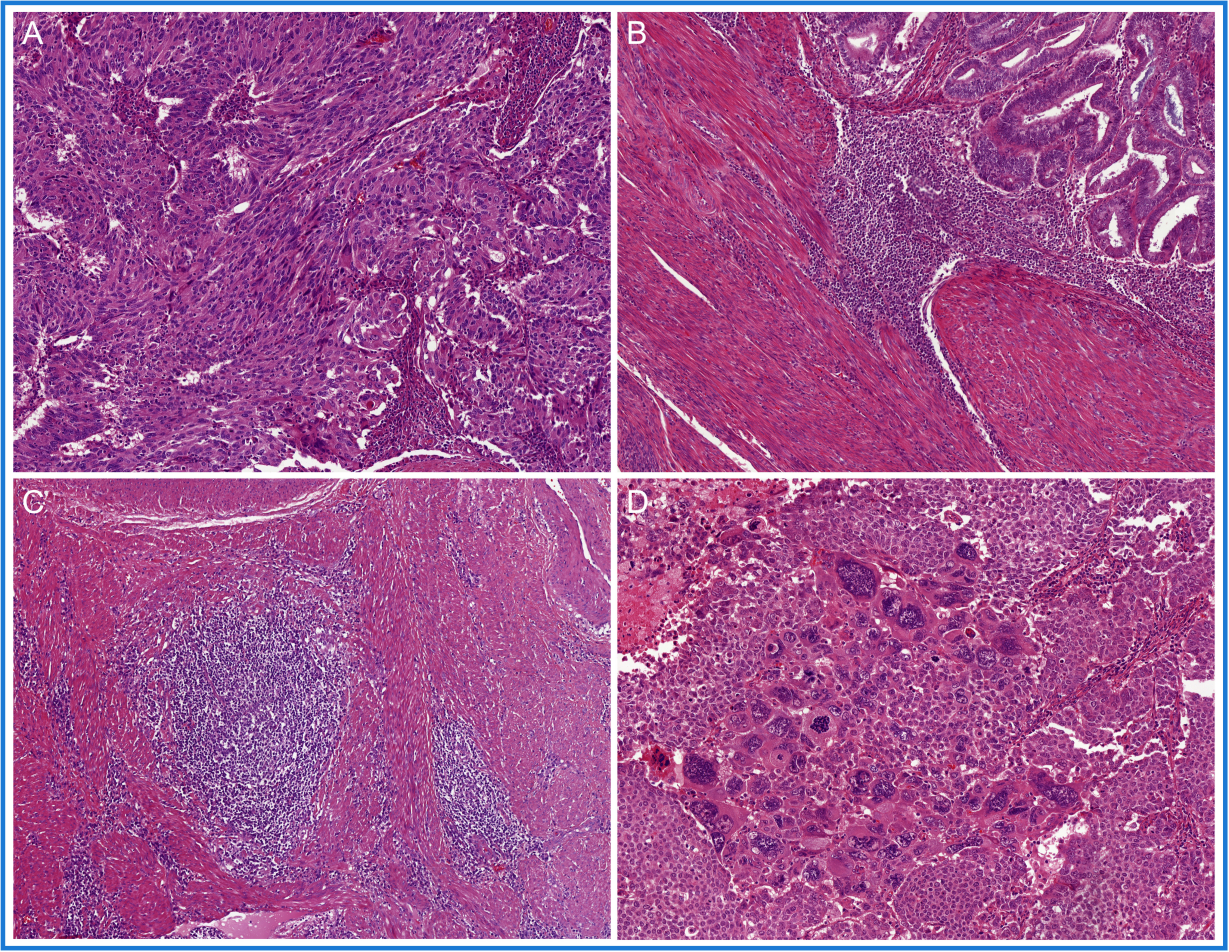
A selection of prototypical morphological features found in *POLE-*mutated endometrial cancer (*POLE*mut EC): **(A)** at least 50% solid growth; **(B)** hyperchromatic tumor giant cells; **(C)** a dense peri-tumoral and intra-epithelial infiltrate of lymphocytes; and **(D)** tertiary lymphoid structures (TLS).

Pathogenic mutations in the exonuclease domain of *DNA polymerase epsilon* (*POLE*) in EC, *POLE*mut EC, were first described by Church et al. (61) and quickly thereafter by the TCGA (5). Mutations in *POLE* result in a defective proofreading domain during DNA leading-strand replication, yielding a very high mutation burden and increased neoantigen load. In these original publications, only a limited number of non-endometrioid EC were tested, and these did not carry *POLE* mutations. This led to the assumption that *POLE* mutations could only occur in endometrioid-type EC. However, subsequent larger studies challenged this idea by showing that *POLE* mutations can be identified in non-endometrioid carcinomas, including uterine carcinosarcomas, serous carcinomas, clear cell carcinomas, and un-/dedifferentiated carcinomas, albeit in low frequencies (14, 62–65). A search for a *POLE*mut EC-specific phenotypic trait resulted in the identification of specific morphological features (**Figure 3**): first, approximately two-thirds of *POLE*mut EC show at least 50% solid growth (also referred to as FIGO grade 3) (66, 67), and the glandular component, if present, usually consists of glands with smooth luminal borders without hobnailing (66); second, hyperchromatic (multi-nucleated) tumor giant cells scattered throughout the solid sheets of tumor cells have been described as a recurring feature (66, 68); third, a dense peri-tumoral and intra-epithelial infiltrate of lymphocytes is frequently noted, likely the phenotypic response of its high mutational load (66, 67, 69–71); finally, a more recent addition to these features is the presence of (often numerous) tertiary lymphoid structures (TLS) within the myometrial wall of *POLE*mut EC (72–74).

To date, there is no single immunohistochemical stain that can serve to identify *POLE*mut EC, and only few studies have studied specific markers (75). Among the IHC stains commonly used in diagnostics, abnormal staining for MMR proteins is present in about 20% of *POLE*mut EC (72). Mutant-type abnormal p53 staining can be identified in 12%–30% of *POLE*mut EC (5, 7, 8), but no specific morphological substrate has been detected in this subset of cases (8). Among the *POLE*mut EC with secondary p53 abnormality, subclonal/regional mutant-type overexpression of p53 is a relatively common finding (7). Sequencing of the exonuclease domain of *POLE* thus remains required to accurately identify *POLE*mut EC, since morphology and adjunct studies alone are insufficient.

### Mismatch repair-deficient endometrial cancers

**Figure 4:**
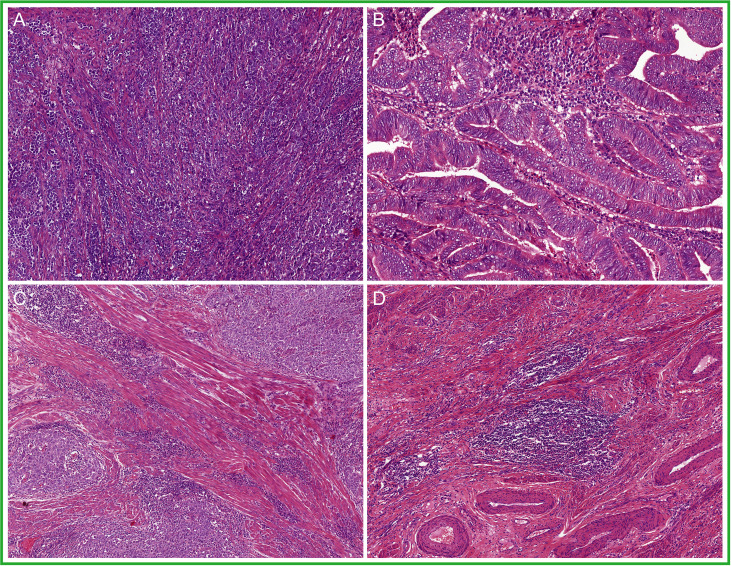
A selection of prototypical morphological features found in mismatch repair deficient endometrial cancer (MMRd EC): **(A)** solid growth; **(B)** glandular architecture; **(C)** a dense to moderate peri-tumoral and intra-epithelial infiltrate of lymphocytes; and **(D)** tertiary lymphoid structures (TLS).

Damage in the DNA mismatch repair (MMR) pathway leaves unrepaired post-DNA replication errors. Thus, the phenotype of MMR-deficient EC (MMRd EC) much like *POLE*mut EC is most likely shaped by the biological consequences of a high mutational load, leading to a similar morphological representation (**Figure 4**). In fact, several studies have described the abundance of stromal and intra-epithelial lymphocytes and, more recently, the presence of TLS in MMRd EC (69, 70, 72, 73, 76), yet in a somewhat lower abundance than in *POLE*mut EC (69, 72). Furthermore, endometrioid-type EC is the dominant histological subtype of MMRd EC (5, 15, 17–19, 77, 78). Endometrioid EC with solid growth (FIGO grade 3) is relatively more frequent in MMRd EC than in NSMP EC but less frequent than in *POLE*mut EC (5, 14, 15, 18). However, a variety of other non-endometrioid histological subtypes have also been reported within the MMRd EC subclass. For example, one recent study identified MMR deficiency in uterine carcinosarcomas and interestingly noted that their epithelial components often had an endometrioid morphology (79). A small proportion of MMRd EC has also been observed with a serous or clear cell morphology (14, 18). This serous-like phenotype was found not to be associated with acquired *TP53* mutations in these MMRd tumors (8). Instead, there is some indication that MMRd EC with serous-like morphology is more often seen in MMRd EC with underlying germline mutations (Lynch syndrome associated); however this, observation needs to be validated (62, 80, 81). Interestingly, about two-thirds of the un-/dedifferentiated EC have been shown to be MMR deficient (82). Finally, for yet unknown reasons, multiple reports described an association between the presence of lymphovascular space invasion (LVSI) and MMR deficiency in EC (15, 77). Hence, morphology alone is insufficient to accurately detect and classify MMRd EC.

After excluding pathogenic *POLE* mutations, immunohistochemical staining of the four MMR proteins is therefore used to identify MMRd EC. Approximately one quarter of all EC show loss of expression of one of the MMR proteins. The most common combination (about 70%) in MMRd EC is loss of MLH1 and PMS2 expression, which is usually caused by promotor hypermethylation of the *MLH1* gene. The remaining cases show loss of protein expression in other combinations, namely, single loss of MSH6, single loss of PMS2, or MSH2/MSH6 loss, of which about 10% is Lynch syndrome associated (80). p53 abnormal staining can be seen in 10% of MMRd EC (72), of which approximatively three quarters show p53 subclonal mutant-type overexpression (7).

### p53 abnormal endometrial cancers

**Figure 5:**
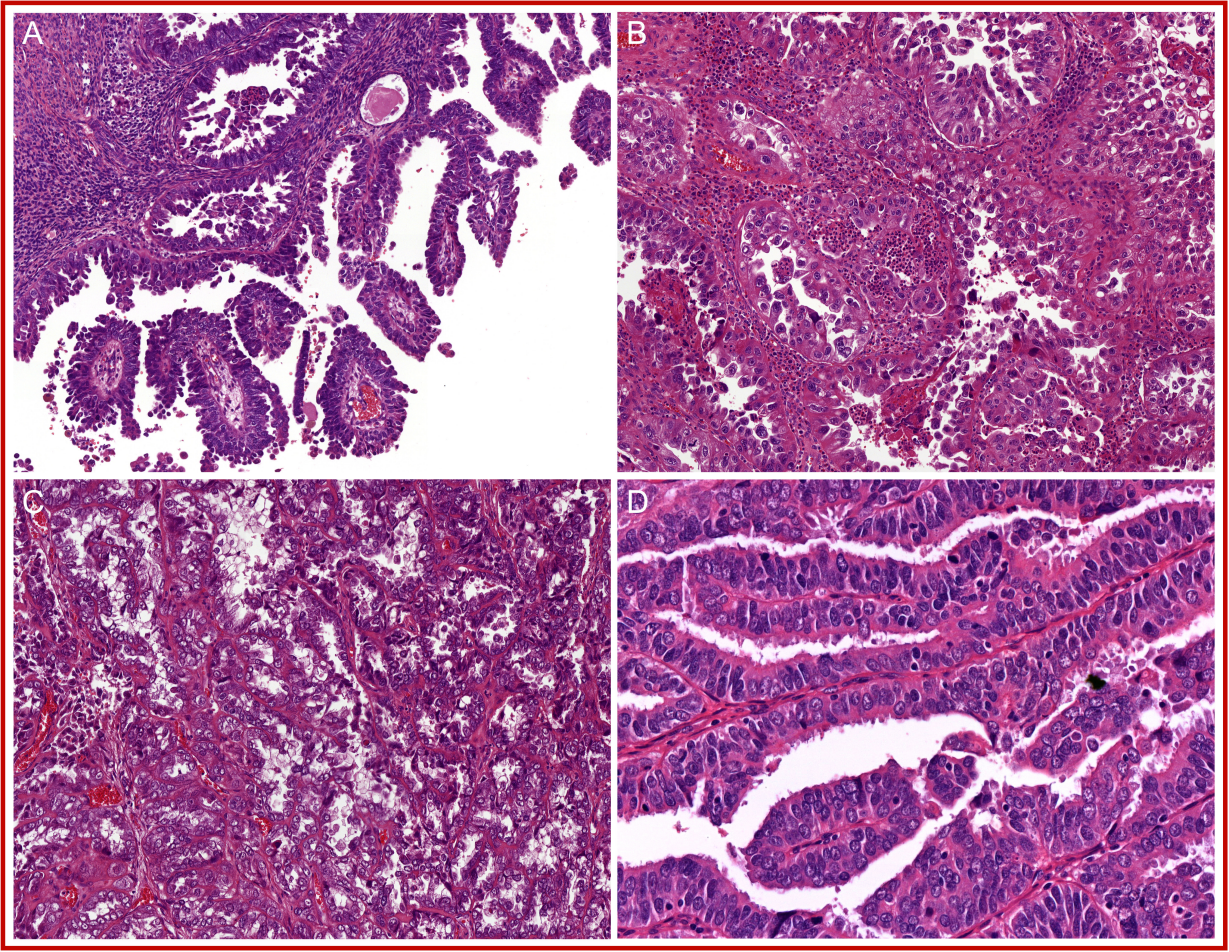
A selection of prototypical morphological features found in p53 abnormal endometrial cancer (p53abn EC): **(A)** (micro-)papillary glandular architecture; **(B)** glands with ragged luminal surface; **(C)** brisk mitotic activity; and **(D)** strong nuclear atypia.

The prototypical p53 abnormal endometrial cancers (p53abn EC) has a classic serous histology with a (micro-) papillary or (pseudo-) glandular architecture. The papillae or glands are lined by a single layer of tumor cells with strong cytonuclear atypia resulting in a ragged luminal surface (**Figure 5**). Furthermore, a brisk mitotic activity is consistently found (14, 18, 78, 83–85). The p53abn EC molecular subgroup, however, has a broader histological spectrum, as it also includes uterine carcinosarcomas (78), clear cell carcinomas (14, 18), and FIGO grade 3 endometrioid carcinomas (5, 14, 18, 84, 85). Intriguingly, some studies described that p53abn EC can also present with low-grade endometrioid morphology (15, 70, 84). Whether this observation is true, or that these cases rather represent misclassified pseudo-glandular serous endometrial carcinomas, remains to be determined. The low abundance of tumor-infiltrating lymphocytes and lack of TLS are other histological features that differentiate p53abn EC from MMRd EC and *POLE*mut EC (69, 70, 72).

p53abn ECs, per definition, are MMR proficient and *POLE* wild type and displays one of the well-described mutant-like immunohistochemical p53 staining patterns (9). This includes abnormal p53 nuclear overexpression in 65%, abnormal null-mutant pattern in 13%, or cytoplasmic p53 overexpression (6, 7). In addition, strong and diffuse positive membranous Her2Neu staining (3+), found in 20%–25% of p53abn EC, may be p53abn subclass specific (7, 86).

### No specific molecular profile endometrial cancers

**Figure 6:**
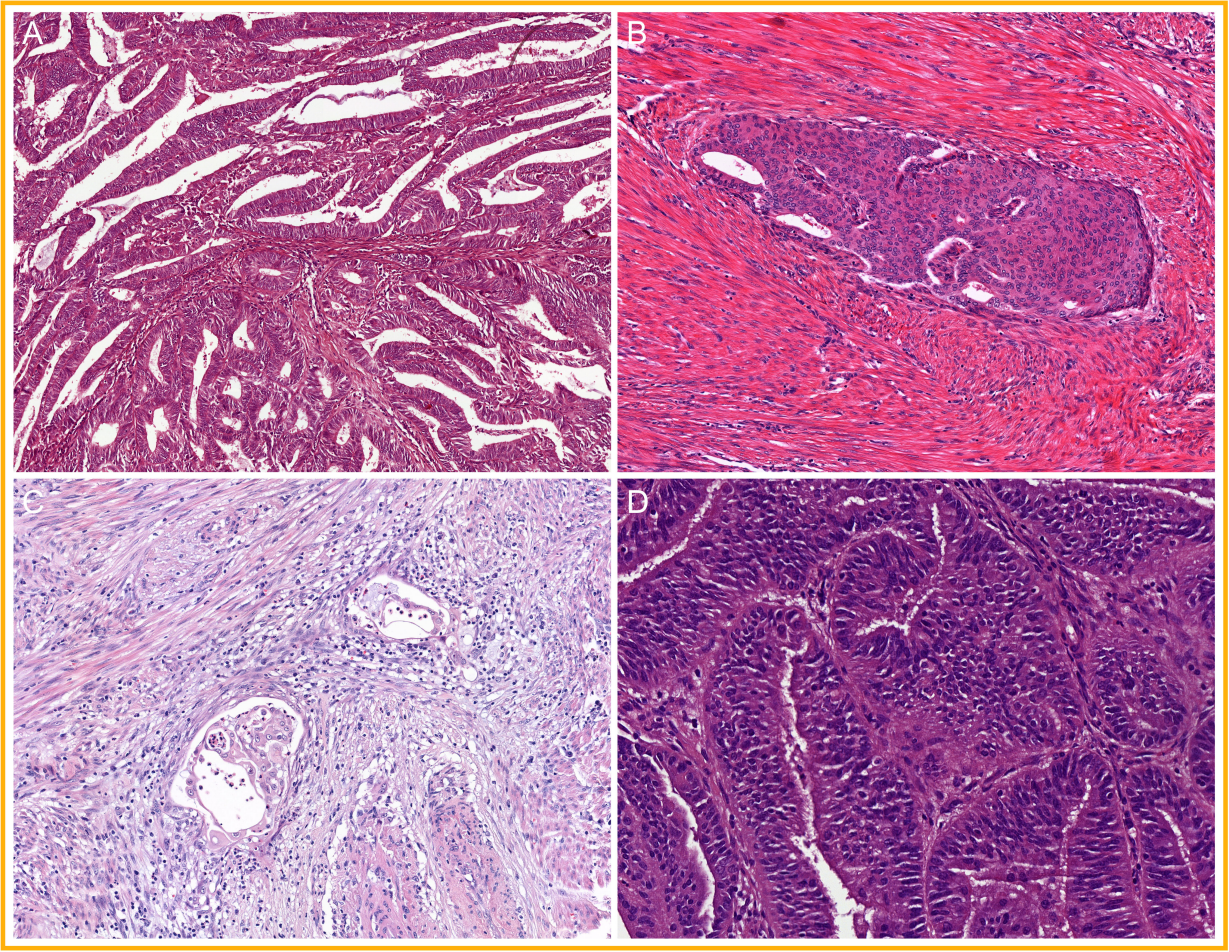
A selection of prototypical morphological features found in non-specific molecular profile endometrial cancer (NSMP EC): **(A)** glands with smooth luminal borders; **(B)** squamous differentiation; **(C)** microcystic elongated and fragmented (MELF) type of invasion; and **(D)** mild nuclear atypia.

The group of EC that does not carry a pathogenic *POLE* mutation is MMR proficient and shows wild-type expression of p53 is currently referred to as “no specific molecular profile” (NSMP) EC. The majority shows a predominant glandular proliferation in which the glands have smooth luminal borders and the nuclei have mild to moderate atypia (FIGO grades 1 and 2) (5, 15, 17, 18, 78). A subset of these low-grade NSMP EC present (morular) squamous differentiation to a varying degree, a distinct morphological feature that has been linked to underlying *CTNNB1* mutations (87–90). In addition to these prototypical features (**Figure 6**), approximately 20% of the low-grade NSMP EC present with a specific type of invasion, referred to as “microcystic elongated and fragmented” (MELF) type of invasion (91–93), which is rarely seen outside NSMP EC. A lower abundance of TILS and TLS in the NSMP EC group than in MMRd EC and *POLE*mut EC has also been reported (69, 70, 72). Finally, non-endometrioid or high-grade NSMP ECs are uncommon but have been described (14, 17–19, 78).

Emerging data suggest that NSMP EC may be further stratified into two groups with a distinct prognosis based on hormone receptor expression status (94). Although the majority of NSMP EC shows high expression of estrogen receptors (ER alpha) and progesterone receptors (PR A/B), a notable subset of approximately 10% of the NSMP EC show complete lack of ER and PR expression. Interestingly, this subgroup is enriched with non-endometrioid morphology, particularly clear cell morphology. It is also conceivable that the recently described mesonephric-like endometrial carcinomas and the gastric-type endometrial carcinomas fall in this group of hormone-receptor-negative NSMP EC (78).

## The current role of morphology within the molecular EC classification

All these outlined human-identified morpho-molecular correlates are presently insufficient to accurately predict molecular class on H&E features only, and no exclusive phenotypic trait has been identified for any of the molecular classes. The observed histopathological heterogeneity within defined molecular classes clearly challenges the role of morphology in the context of the molecular EC classification. Morphological information may still refine prognosis within a specific molecular context such as histological subtype and grade in NSMP EC (95) or dense immune infiltrate or presence of TLS in MMRd EC (69, 70). Yet, at the same time, some morphological features may arguably no longer carry prognostic value in some molecular subgroups. For instance, evidence showed that a broad range of histological subtypes and grades can be found in *POLE*mut EC and p53abn EC while carrying distinct genomic alterations and having excellent and poor prognosis, respectively (5, 14, 15, 17–20). Likewise, the prognostic relevance of other morphological features is under investigation such as the presence of LVSI within *POLE*mut EC and MMRd EC (15, 66, 77) and the lymphocyte density in NSMP EC and p53abn EC (69, 70, 72).

Implementation of the molecular EC classification is a step forward, but it is questionable whether histological features have become completely obsolete. Morphological features may still contain pertinent information beyond the molecular classification such as additional prognostic indicators. It is, however, becoming increasingly complex for pathologists to distinguish relevant morphological subtleties in EC diagnostics. In this context, DL models may be capable of learning relevant morphological features in association with molecular alterations on digitized H&E-stained EC tumor slides. DL-based research may show that further refining of the EC classification is possible by accurately combining histological and molecular data (**Figure 2**).

## Deep learning can recognize phenotypes of mutations on H&E tumor slides

Landmark studies have recently provided the proof of principle for the prediction of genetic mutations from H&E whole slide images by DL in several types of cancer (28–52, 54, 55), albeit more frequently in colorectal cancer (29–37) and breast cancer (35, 38–44). For example, the feasibility of predicting *TP53* mutation status has been explored across breast, colorectal, lung, stomach, pan-gastrointestinal, bladder, and liver cancer (36, 48, 51, 52, 55). In breast cancer, prediction of hormone receptor status (38, 39, 42) and homologous recombination deficiency (35, 40) has also been investigated. A common task for DL has also been the prediction of microsatellite instability, particularly in colorectal cancer (29, 31–36) and gastrointestinal cancer (40, 45, 46). Across all these studies, encouraging performance was measured with the area under the receiver operating characteristic (ROC) curve (AUC) on some external test sets above 0.80 (29, 29, 32, 34, 36–39, 45, 48). Although sensitivity and specificity may be, at this date, insufficient for end-to-end clinical implementations, this is a proof of concept that genotype–phenotype correlations can be identified using DL on H&E-stained tumor slides. Sirinukunwattana et al. (37) tackled a more complex task: a DL model predicted the four-class consensus molecular subtypes (CMS) of colorectal cancer, obtaining a 0.84 AUC on the TCGA external test set (N=431 slides from 430 patients). Among these DL-based studies, one research angle that has got some particular interests is explaining the DL predictions, frequently referred to as a “black box” and deriving human-interpretable features (31, 36, 37, 44). For instance, this can be done by extracting subregions of the input slide image that were assigned strong weights by the DL model to molecularly classify a given case. The visual assessment of these regions of interest can be used to reveal relevant morpho-molecular correlates, although this may not always provide human-interpretable visual information. Another approach can be to correlate the DL predictions with clinicopathological data.

Similar DL performance and interpretability still need to be shown in large cohorts of EC. To date, only Wang et al. (53) and Hong et al. (54) have trained supervised binary classification models from H&E-stained EC tumor slides and labels publicly available from the TCGA and Clinical Proteomic Tumour Analysis Consortium (CPTAC) datasets. Wang et al. (53) limited the predictions to high microsatellite instability (MSI) on the TCGA (N=516 of which 128 MSI patients) and obtained an AUC of 0.73 on 25% hold-out internal test set. Hong et al. (54) predicted various mutations and each of the four TCGA-derived molecular EC classes separately. To this end, they reached an AUC of 0.66 for *POLE* mutation (N=7 *POLE*-mutated patients), 0.76 for MSI (N=25 MSI patients), 0.87 for copy-number high (N=20 copy-number high patients), and 0.65 for copy-number low (N=43 copy-number low patients) on the CPTAC external test set. Additionally, they obtained an AUC of 0.77 for the prediction of the *TP53* mutational status (N=30 *TP53*-mutated patients). Although both studies represent first proofs of concept of predicting genetic mutations from H&E slide images that can be expanded to EC, the test sets remained relatively small and did not reflect the heterogeneity of histological subtypes and FIGO grades known to be present in each molecular class. Specifically, with a few non-endometroid samples included in the TCGA (5) and CPTAC (96), the applicability to large cohorts of non-endometrioid EC remains unknown. In addition, the authors limited the scope of mutation prediction to binary classification tasks. Thus, to date, leveraging DL to predict the four-class molecular EC classification and deriving human-interpretable morpho-molecular correlates, have yet to be explored. Finally, the DL models used in both studies first divided the slide images into tiles, which is a standard computational method in the field, and then classified each tile individually with labels assigned at the slide-level. The tile-level classification may introduce training noise because morphological information in a given tile does not always correlate with the given true slide-level label. Hong et al. (54) have also acknowledged this architectural limitation, reporting that non-tumor tiles were often given inconclusive prediction scores. The discrepancy between tile and slide-level classification labels remains a well-known challenge in the field, which has started transitioning to more state-of-the-art DL architectures promising better performance (97–100).

## Integrating deep learning into the current molecular EC classification

The gynecopathological community has started exploring how morphology-based information could be used to aid the molecular EC classification for an optimized risk-stratification strategy. Assistance to accurately weigh both histopathological and molecular variables would be welcome, as the number of relevant variables is steadily increasing (101). The innate strength of DL technology for the analysis of multi-modal datasets including both image and molecular information suggests that DL could aid in the refinement of the current morpho-molecular classification of EC.

EC-specific DL tasks could range from predicting one specific molecular alteration to predicting the complete four-class molecular classification from standard H&E images or from a combination of H&E images and special stains. To achieve this, the input data for DL models are digitized whole slide histopathology images of EC with the associated EC molecular classes. Importantly, such models achieve an incrementally increasing performance with the size and quality of the available datasets, ground-truth annotations, and advances in DL technology. Furthermore, such models can be purposely designed to generalize to previously unseen datasets and can be run in a cloud environment, potentially enabling broad access to AI-guided classification in future pathology.

This remarkable technological paradigm chance could support an increase in the fidelity of EC patient diagnosis, prognostic, and predictive classification impacting the whole diagnostic process and treatment decision-making. Theoretically, if a DL model predicts the four-class molecular EC classification at near perfect high specificity and sensitivity, then one could envision that DNA sequencing and immunohistochemistry would only be required for confirmatory testing, if at all. If such a DL model is also shown to be generalizable to external cohorts, EC patients could be molecularly classified using only digitized H&E-stained tumor slides. In this scenario, this automated tool would be clinically relevant by (i) providing a cost-effective alternative to expensive molecular testing without the need of additional tissue and (ii) speeding the diagnosis process up and advancing treatment initiation, which, in a real-world practice, can be delayed by weeks with next-generation sequencing. Until a clinical-grade accuracy of such model has been achieved, alternative and less complex DL tasks can be taken forward to support EC patient management. In fact, a binary predictive model trained toward one single specific molecular alteration may yield a higher sensitivity and specificity than a four-class model and could subsequently serve as a pre-screening tool. In the field of EC, pre-identifying p53abn EC in a population of low-risk EC may potentially be used to identify those few patients with a poor prognosis for confirmatory testing (15). Similarly, it may be possible to identify the aggressive subset of NSMP EC that lack hormone receptor expression (94). Furthermore, preselecting cases that would further require *POLE* testing given MMR-IHC and p53-IHC would be particularly supportive and cost saving in high-risk ECs, as treatment de-escalation of *POLE*mut EC with good prognosis is currently being explored (5, 14–17, 19, 65, 70).

An avenue by which DL has currently probably the biggest role is as a research tool combined with gynecopathological expertise. Several studies showed (36, 37, 44) that after training an EC-specific DL model, image-based information associated with EC molecular alterations could be visually extracted and reviewed by gynecopathologists. From there on, the morpho-molecular correlates that are outlined in this review may be confirmed, but the DL model may also reveal morpho-molecular features that have so far not struck the attention of human observers. Increasing knowledge about the morphology of the molecular classes will help to understand the biological processes and dynamics of tumor–host interaction in the tumor microenvironment. The prognostic value of the identified morpho-molecular features can be subsequently explored, which may open new doors to prognostic refinement in EC.

With increasing availability of digitalization aids such as cloud computing and resources, DL-driven diagnostic tools could be made available worldwide as an additional inexpensive, if not free, resource without the need of local hardware or knowledge (102). Particularly, users without access to scanners would only need to generate slide images using microscope cameras or even existing mobile phones for diagnostic classification in a central expert center. However, high-quality slide images may remain a limitation to the applicability in low-income countries and country-specific regulations on patient data transfer.

## Discussion

In the past four decades, the classification of EC has evolved from a histology-based to a molecular system. The recent integration into guidelines indicates the increasing prognostic value of the four-class molecular classification over morphology in EC (1, 2), yet the integrated management with former histopathological variables is still a challenge in the diagnostic routine (9). As a result, questions have been raised about the relevance of these histopathological features and the role of morphology beyond the molecular EC classification. Now, given the four molecular EC classes, a number of studies have described some distinct morphological characteristics, but they remain insufficient to achieve accurate classification (66–70, 72, 73, 76, 83), and the pathologist’s eyes are not sufficiently trained to spotlight them. In fact, this review stresses the difficulty in weighing image-based information in relation to the current four molecular EC classes. First, each molecular subclass shows heterogeneity for histological subtype, FIGO grade, and associated microscopic features. Second, many microscopic features appear to be non-exclusive, for instance the presence of high levels of immune cells between *POLE*mut *EC* and MMRd EC (69, 70). Lastly, some morphological traits are detectable at different magnifications and growth patterns, and nuclear atypia within p53abn EC is one example.

In the quickly progressing research domain of computer science, DL has demonstrated a well-known capability to work with high-dimensional and multi-modal datasets, up to learn phenotype–genotype correlates from highly complex and extra-large digitized tumor slides (29–52, 54, 55). Hence, future DL-based breakthroughs have legitimate potential to resolve the current dilemma between molecular and histopathological variables or even support EC patient management for pre-screening and decision-making on treatment, ultimately impacting EC diagnostics and patient care as a whole. As for today, an urgent assignment given to DL technology in combination with gynecopathological expertise is bringing to the surface the clinical relevance of each morphological feature associated with the four molecular EC subclasses, while improving morphological and biological understanding of the genomic EC alterations. Combining the strengths of molecular-, clinical-, and DL-based information may refine the EC classification to reach optimal prognostication and prediction for our future EC patients.

## Author contributions

SF wrote sections of the review. All authors contributed to manuscript revision, read, and approved the submitted version.

## Funding

This work was supported by an unrestricted grant by the Hanarth Foundation.

## Conflict of interest

VK: Research grants from Swiss Federal Institute of Technology Strategic Focus Area: Personalized Health and Related Technologies PHRT, the Swiss National Science Foundation and Promedica unrelated to the current works. NH: Unrestricted research grants unrelated to the current work from the Dutch Cancer Society and Varian. TB: Research grants from the Dutch Cancer Society unrelated to the current work.

The remaining authors declare that the research was conducted in the absence of any commercial or financial relationships that could be construed as a potential conflict of interest.

## Publisher’s note

All claims expressed in this article are solely those of the authors and do not necessarily represent those of their affiliated organizations, or those of the publisher, the editors and the reviewers. Any product that may be evaluated in this article, or claim that may be made by its manufacturer, is not guaranteed or endorsed by the publisher.
